# Microbial colonization of basaltic glasses in hydrothermal organic-rich sediments at Guaymas Basin

**DOI:** 10.3389/fmicb.2013.00250

**Published:** 2013-08-27

**Authors:** Nolwenn Callac, Céline Rommevaux-Jestin, Olivier Rouxel, Françoise Lesongeur, Céline Liorzou, Claire Bollinger, Antony Ferrant, Anne Godfroy

**Affiliations:** ^1^Laboratoire de Microbiologie des Environnements Extrêmes UMR 6197, Université de Bretagne Occidentale, UEB, IUEMPlouzané, France; ^2^Laboratoire de Microbiologie des Environnements Extrêmes UMR 6197, IfremerPlouzané, France; ^3^Laboratoire de Microbiologie des Environnements Extrêmes UMR 6197, CNRSPlouzané, France; ^4^Domaines Océaniques UMR6538, IUEM, Université de Bretagne OccidentalePlouzané, France; ^5^Laboratoire Géobiosphère Actuelle et Primitive, CNRS, IPGP, Sorbonne Paris Cité, Univ Paris Diderot, UMR 7154Paris, France; ^6^Laboratoire de Géochimie et de Métallogénie, IfremerPlouzané, France; ^7^IUEM, Université de Bretagne Occidentale, UMS 3113Plouzané, France; ^8^Unité Recherches et Développements Technologiques, IfremerPlouzané, France

**Keywords:** colonization module, basalt alteration, Guaymas basin, organic-rich sediment, hydrothermal systems

## Abstract

Oceanic basalts host diverse microbial communities with various metabolisms involved in C, N, S, and Fe biogeochemical cycles which may contribute to mineral and glass alteration processes at, and below the seafloor. In order to study the microbial colonization on basaltic glasses and their potential biotic/abiotic weathering products, two colonization modules called AISICS (“Autonomous *in situ* Instrumented Colonization System”) were deployed in hydrothermal deep-sea sediments at the Guaymas Basin for 8 days and 22 days. Each AISICS module contained 18 colonizers (including sterile controls) filled with basaltic glasses of contrasting composition. Chemical analyses of ambient fluids sampled through the colonizers showed a greater contribution of hydrothermal fluids (maximum temperature 57.6°C) for the module deployed during the longer time period. For each colonizer, the phylogenetic diversity and metabolic function of bacterial and archaeal communities were explored using a molecular approach by cloning and sequencing. Results showed large microbial diversity in all colonizers. The bacterial distribution was primarily linked to the deployment duration, as well as the depth for the short deployment time module. Some 16s rRNA sequences formed a new cluster of *Epsilonproteobacteria*. Within the Archaea the retrieved diversity could not be linked to either duration, depth or substrata. However, *mcrA* gene sequences belonging to the ANME-1 mcrA-guaymas cluster were found sometimes associated with their putative sulfate-reducers syntrophs depending on the colonizers. Although no specific glass alteration texture was identified, nano-crystals of barite and pyrite were observed in close association with organic matter, suggesting a possible biological mediation. This study gives new insights into the colonization steps of volcanic rock substrates and the capability of microbial communities to exploit new environmental conditions.

## Introduction

Alteration of the oceanic crust by seawater is one of the most important processes controlling the global fluxes of many elements at mid-oceanic ridges and ridge flanks (e.g., Staudigel and Hart, 1983; Wheat and Mottl, 2000) and the mineralogical and chemical composition of the aging oceanic crust (Alt, 1995). Since sub-seafloor basaltic crust represents the largest habitable zone by volume on Earth, microbes may play a significant role in the alteration process (Bach and Edwards, 2003). Microorganisms exploiting these reactions are known from basalt exposed at the seafloor, where the oxidation of reduced sulfur (S) and iron (Fe) compounds from basalt with dissolved oxygen and nitrate from seawater supports high microbial biomass and diversity (Mason et al., 2008; Santelli et al., 2008a; Orcutt et al., 2011b). It has been also demonstrated that seafloor basalts harbor diverse microbial communities either on the rock surfaces (epilithic microorganisms) or inside the rocks (endolithic microorganisms; Mason et al., 2007; Santelli et al., 2009).

Seafloor hydrothermal systems are also complex environments with highly diverse and active microbial communities (Schrenk et al., 2003; Edwards et al., 2005; Nakagawa et al., 2006; Page et al., 2008; Flores et al., 2011) fueled by steep physical and chemical gradients in the mixing zone between oxygenated cold seawater and reduced metal-rich high temperature hydrothermal fluid. Likewise, seafloor hydrothermal chimneys and hydrothermally-affected sediments provide specific habitats hosting a wide range of microorganisms involved in key biogeochemical reactions related to carbon, sulfur, nitrogen, and iron cycles (Burggraf et al., 1990; Kashefi et al., 2002; Teske et al., 2002; Dhillon et al., 2003; Francis et al., 2007; Byrne et al., 2009; Biddle et al., 2012; Bowles et al., 2012). Hence, microorganisms interact with their environment in many ways, and, in turn, could affect fluid composition, and promote mineral dissolution or precipitation (Edwards et al., 2003a, 2005; Houghton and Seyfried Jr, 2010). Evidence for microbial alteration of basaltic glasses is also increasing, and includes the alteration textures of volcanic glass (Furnes et al., 2001; Einen et al., 2006) as well as putative presence of DNA revealed by high C, N, and P contents in altered glass (Thorseth et al., 1992). The light isotopic composition of C and S in altered basalts also demonstrates potential organic C cycling and sulfate reduction within volcanic basement (Furnes et al., 2001; Rouxel et al., 2008b).

Hydrothermally heated sediments covering oceanic basalts are present in the Guaymas Basin, one of the semi-closed basins of the Gulf of California (Mexico). The Guaymas Basin is covered by a thick layer of organic and diatomaceous-rich sediments (100–500 m) due to a high sedimentation rate (up to 2 mm per year) and biological productivity in the upper ocean (Simoneit and Lonsdale, 1982; Von Damm et al., 1985b; De La Lanza-Espino and Soto, 1999; Dean et al., 2004). In the Southern Trough area, where crustal accretion takes place (Lonsdale and Becker, 1985), the seafloor is exposed to high-temperature hydrothermal activity. The circulation of hydrothermal fluids results in both the formation of sulfide and carbonate-rich chimneys and profoundly affects sediment geochemistry. Diagenetic interactions between the ascending hydrothermal fluids and sediments result in the pyrolysis of organic matter and precipitation of metal-sulfide minerals in subsurface (e.g., pyrrhotite FeS). Products of pyrolysis include light hydrocarbons, short-chain organic acids, particulate organic matter, ammonia and methane (Welhan, 1988; Martens, 1990) which provide unique conditions for sustaining uncommon and diverse microbial life (Teske et al., 2002). Likewise, microbial communities within microbial mats at Guaymas Basin have been extensively studied in term of their physiological and phylogenetical diversity, using both cultural and molecular approaches (Teske et al., 2002; Dhillon et al., 2005; Holler et al., 2011; Biddle et al., 2012; Bowles et al., 2012; Mckay et al., 2012).

The colonization of mineral substrates in hydrothermal environments or their vicinity has been already studied using diverse approaches in order to assess both prokaryotic and micro-eukaryotic diversity. Many microbial colonization systems (e.g., vent caps, TRAC, ISCS, vent catheters, growth chamber, thermocouples) were previously deployed on various hydrothermal areas (Reysenbach et al., 2000; Corre et al., 2001; Takai et al., 2003; Alain et al., 2004; Higashi et al., 2004; Page et al., 2008; Rassa et al., 2009). Those studies generally showed that the *Epsilonproteobacteria* were dominant, and that the microbial diversity can vary both in terms of structure and size, depending on environmental conditions, mineral substrate composition, and deployment duration. More recently, rock substrates were deployed directly in boreholes (Orcutt et al., 2010, 2011a; Edwards et al., 2011) using the FLOCSs (Flow-Trough Osmo Colonization Systems). So far, microbial or/and abiotic alteration of basaltic glasses were investigated at low (i.e., 3–4°C; Mason et al., 2007; Santelli et al., 2009) to medium temperatures (i.e., 40 and 60°C; Orcutt et al., 2010, 2011a) in organic-matter poor volcanic environments. However, little is known about microbial colonization processes and basaltic glass alteration under hydrothermal conditions and in an organic-matter rich system, especially in term of the carbon and energy sources for microbial life and impact on basaltic glass alteration. Here, the AISICS “Autonomous *in situ* Instrumented Colonization System” containing basaltic substrata was deployed for 8 and 22 days into the sediments underlying microbial mats and exposed to hydrothermal conditions in the Guaymas Basin. Since basaltic glass substrates exposed to *in situ* conditions may be affected by both biological and inorganic (i.e., fluid/rock) interactions, colonization experiments were systematically performed in the presence of abiotic controls. The microbial diversity of the samples was analyzed using 16S rRNA and functional gene sequencing, and fluids were recovered to determine their chemical composition. Moreover, glass alteration and secondary mineral precipitation were investigated under both biotic and abiotic conditions.

## Materials and methods

### Site description

**Figure 1:**
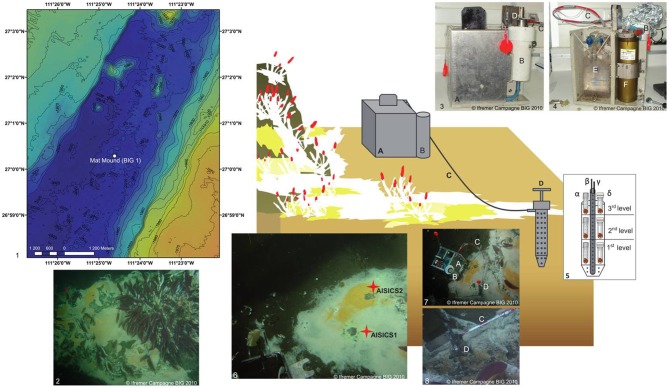
**Schematic diagram illustrating the deployment of the *in situ* AISICS module at Mat Mound site. (1)** Bathymetric map showing the location of Mat Mound site in the Southern Trough of the Guaymas Basin; **(2)** Mat Mound site exhibiting microbial mats and macro-fauna dominated by *Riftia* tub tube worms (Siboglinidae); (photo taken with the submersible Nautile during the BIG cruise, Dive 1745); **(3)** AISICS module covered by its lid before its deployment; **(4)** AISICS module without its lid before its deployment; **(5)** Diagram illustrating the internal structure of the incubator with biotic (α) and abiotic (β) mini-colonizers distributed per floor; the central titanium sheath containing the Micrel temperature sensor (γ) and the fluid sample probe (δ) hosted in a titanium sheath are placed in the middle of the incubator and are connected to the instrumented base; **(6)** The deployment site of AISICS1 and 2; **(7)** The deployed AISICS1 (photo was taken with the submersible Nautile during the BIG cruise, Dives 1745); **(8)** The deployed AISICS2 (photo was taken with the submersible Nautile during the BIG cruise, Dives 1763); (A) instrumented module; (B) cylindrical insulated chamber; (C) sampling pipes and temperature probe; (D) incubator; (E) sampling pouches, and (F) electronics.

Deployments were conducted by the research submersible *Nautile* (Ifremer) during the BIG (Biodiversité et Interactions à Guaymas) oceanographic cruise (RV *L'Atalante*) that took place in the Guaymas Basin in June 2010. AISICS deployments were performed at the Mat Mound site (N27°00.388, W111°25.471; 2004 m depth, BIG1 Marker) on the Southern Trough (Figure 1). This site consists of a small sulfide- and carbonate-rich active hydrothermal mound emerging above the sediment at the seafloor. The mound and surrounding sediments are covered by thick, white and orange microbial mats. The macrofauna is dominated by dense *Riftia* worm bushes at the top of the mound, and *Alvinellids* and *Polynoids* around the mound (Figure 1). The choice of this site was guided by the occurrence of abundant white and orange microbial mat. The colonizers were deployed within a 20 cm^2^ area located on the edge of a white microbial mat at the base of the mound. Temperatures of 36.5, 68, 84.5, and 103°C were measured at 10, 20, 30, and 40 cm depth below seafloor, respectively. The deployment and recovery of the AISICS module were carried out one after the other, in order to minimize sediment and fluid flow disturbance.

### Description of the autonomous *in situ* instrumented colonization system

The **AISICS** system is an autonomous instrumented microbial colonizer. It consists of the incubator itself and the instrumented module (Figure 1) (Sarrazin et al., 2006). The incubator is a titanium cylindrical chamber, perforated by numerous apertures 0.5 cm in diameter. A central titanium sheath, also perforated with 0.5 cm holes, hosts a Micrel™ temperature sensor and a titanium sampling pipe (0.5 cm diameter) both connected to the instrumented module by a 1 m long sampling tube. The AISICS instrumented module contains the electronic control card and battery for the pumping system encased in a watertight cylinder. The temperature probe electronics and four 100 mL sampling bags (PVC pouch, Baxter Clinic) are connected to a four-ways pump device for fluid collection (Sarrazin et al., 2006). The pump speed was set to a low flow rate (3.3 mL min^−1^) in order to minimize environmental perturbation. The insulated chamber was designed for aseptic transportation of the incubator by the means of o-rings at the top and bottom. The temperature probe was computer-encoded before deployment to record the temperature at regular time intervals. The four-way valve and fluid pumping device was also programmed on board to set the trigger time for fluid sampling. Within each AISICS module, a total of 18 mini-colonizers were placed around the central sheath, and stacked over three layers (i.e., six per floor; 4 biotic, 2 abiotic). A perforated Teflon disk separated each layer from the other and allowed fluid circulation through the colonizers, (Figure 1). For the biotic experiments, the mini-colonizers consisted of a set of 2 mL polypropylene microtubes with caps (SX-8G IP-Star® Compact), both perforated with 1 mm holes (Figure 1). For abiotic controls, the mini-colonizers also consist of a set of 2 mL polypropylene microtubes (SX-8G IP-Star® Compact) with the cap replaced by a 0.22 μm filter cellulose membrane (Millipore; Figure 1). The apertures of the incubator, Teflon disk and mini-colonizer tubes and caps, ensure fluid exchange throughout the different compartment of the mini-colonizers.

### Substrata, instrumental setting, and deployment

Synthetic basaltic glasses were prepared using a mixture of pure element oxide and carbonate powder leading after synthesis to typical composition of tholeiitic basalt (with proportion in weight %: SiO_2_, 48.68; Al_2_O_3_, 15.7; CaO, 11.2; MgO, 7.7; FeO, 12.5; Na_2_O, 2.7; K_2_O, 0.2; TiO_2_, 1.39). One batch of synthetic basaltic glass was prepared using ^57^Fe-enriched Fe_2_O_3_ powder obtained from Oak Ridge National Laboratory. Before mixing in agate mortar, powders were dried at 150°C for at least 24 h. Two different furnaces were used to prepare glass beads: a Carbolite™ 1700 muffle furnace with a maximum temperature of 1600°C with manual quenching under ambient atmosphere conditions, and a vertical furnace, mounted at Geomaterials laboratory (Univ. Marne La Vallée, France), with automatic quench system under controlled atmosphere (H_2_ or O_2_). The glass beads were prepared according to the following scheme: a temperature ramp up to 600°C for 30 min to 2h, decarbonation at 600°C for 45 min to 1h, another temperature increase up to 1600°C from 45 min to 3 h, followed by 60 min at 1600°C and immediate quenching.

A sample of natural basaltic glass was obtained by separating the chilled margin of a pillow basalt (sample Bat09-ROC22) from the Mid-Atlantic Ridge recovered during the Bathyluck cruise (2009) at Lucky Strike hydrothermal field. Glass composition (wt%) has been determined: SiO_2_, 51.74; Al_2_O_3_, 14.96; CaO, 12.18; MgO, 8.1; Fe_2_O_3_, 9.95; Na_2_O, 2.28; K_2_O, 0.16; TiO_2_, 1.05; MnO, 0.18; P_2_O_5_, 0.12. All natural and synthetic glasses were crushed in an agate mortar to obtain fragments of less than 2 mm in size. Chips were subsequently cleaned in an ultrasonic bath in ethanol and then air-dried.

Each mini-colonizer was filled with about 0.6 mL of glass fragments, and sterilized by autoclaving during 30 min at 121°C, then by UV for at least 1 h. All titanium parts (i.e., incubator and the central titanium sheath) and Teflon-disks were rinsed five times with deionized water (MilliQ™ 18 mΩ), cleaned up using Desibac HPC® solution, rinsed again with deionized water then with Ethanol 96% and finally UV-treated for at least 1 h. The cylindrical insulated chamber was also cleaned using Desibac HPC®, deionized water, and Ethanol 96% then filled with sterilized seawater prior to deployment.

### AISICS1 and 2

The AISICS1 module was deployed in the sediment at 40 cm depth below a thick white microbial mat (Figure 1). The maximum temperature reached at this depth was measured at 57.6°C over the 22 days of deployment. The AISICS1 mini-colonizers were filled with three different basaltic glass types: two synthetic glasses including one doped with ^57^Fe (noted, respectively, β syn and β syn^*^), and the basaltic glass (noted β nat). Each of the three layers contained 1 biotic mini-colonizer with β syn, 1 biotic mini-colonizer with β syn, 2 biotic mini-colonizers with β nat, 1 abiotic mini-colonizer with β syn and 1 abiotic mini-colonizer with β nat. The temperature measurement frequency was fixed every 30 s. The fluid pumping system was programmed to collect three fluid samples at 48 h intervals.

The AISICS2 module was deployed for 8 days, at the junction between a white and orange microbial mat, at a distance of 5–10 cm from the location of AISICS1 module (Figure 1). Each of the three layers contained two biotic mini-colonizers filled with β nat and two others with β syn^*^ and one abiotic tube for each substrate. Because of the short duration of deployment of this module, the temperature measurement frequency was set for every second and the fluid pumping system was programmed to collect fluids every 48 h after deployment.

### Sample processing

Immediately after on board recovery, each glass sample from the mini-colonizers was aseptically split into five fractions. Two fractions were stored for molecular diversity analysis by freezing one at −80°C and storing the other at −20°C in 96% ethanol. One fraction was stored directly at −20°C for Scanning Electron Microscopy (SEM) and RAMAN spectroscopy analysis; one fraction was fixed for 2 h in 2% formaldehyde (prepared with sterile seawater), rinsed 3 times with sterile seawater and stored in 96% ethanol at −20°C for further Fluorescent *in situ* Hybridization (FISH) experiments and SEM analysis, as the last fraction directly stored in 50% ethanol—phosphate-buffered saline pH 7.2 (PBS) 1× solution (1:1) at −20°C. During processing of the mini-colonizers located in the 3rd level of the AISICS1 module, biotic β nat and β syn^*^ samples were accidently mixed but nevertheless treated, and referred as β mix.

### DNA extraction

Total genomic DNA was extracted from the two fractions of basaltic glasses for molecular diversity analysis, using the FastDNA® Spin Kit for Soil (Bio101 Systems, MP Biomedicals), following the protocol modified by Webster et al. (2003). The DNA extractions of each sample were done independently for each type of storage and the extraction products were then pooled prior to PCR amplification.

### 16S rRNA gene amplification

The 16S rRNA gene was amplified using the specific archaeal or bacterial domain primer combinations of A8F and ARC915R (Casamayor et al., 2000; Kolganova et al., 2002) and E8F and U907R (Lane et al., 1985; Lane, 1991), respectively (Table 1). Both archaeal and bacterial 16S rRNA gene amplification reactions were performed in 50 μl reaction mixtures containing: 10 μl of 5× GO Taq® DNA polymerase buffer (Promega), 5 μl of 25 mM MgCl_2_ solution (Promega), 1 μl of 10 mM dNTPs (Eurogentec), 0.2 μl of each primers at 100 μM and 0.24 μl of 5 U.μl^−1^ GO Taq® DNA polymerase (Promega). All amplifications were conducted in 30 cycles of denaturation at 94°C for 1 min, annealing for 1 min 30 s at 58°C or 52°C for the archaeal or bacterial 16S rRNA gene, respectively, and extension at 72°C for 7 min. All PCR reactions were carried out using a GeneAmp® PCR system 9700 (Applied Biosystems) thermal cycler, and PCR products were visualized using gel electrophoresis.

**Table 1:** **List of the PCR primers used during the study**.

**Primers**	**Target**	**Sequence (5′-3′)**	**Tm°C**	**References**
A8F	Archaeal 16S rRNA	CGG-TTG-ATC-CTG-CCG-GA	58	Kolganova et al., 2002
ARC915R		CTG-CTC-CCC-CGC-CAA-TTC-CT		Casamayor et al., 2000
E8F	Bacterial 16S rRNA	AGA-GTT-TGA-TCA-TGG-CTC-AG	52	Lane, 1991
U907R		CCG-TCA-ATT-CMT-TTG-AGT-TT		Lane et al., 1985
DSR1F	*dsrAB* gene	AC[C/G]-CAC-TGG-AAG-CAC-G	55	Wagner et al., 1998
DSR4R		GTG-TAG-CAG-TTA-CCG-CA		
ME1	*mcrA* gene	GCM-ATG-CAR-ATH-GGW-ATG-TC	50	Hales et al., 1996
ME2		TCA-TKG-CRT-AGT-TDG-GRT-AGT		

### PCR amplification of functional genes

The presence of sulfate-reducers was highlighted with the amplification of *dsrAB* gene targets [coding for the (di)sulfite reductase], with a DSR1F and DSR4R primer combination (Wagner et al., 1998) (Table 1). The presence of methanogens was investigated with the amplification of *mcrA* gene (coding for the alpha subunit of the methyl-coenzyme M-reductase) using ME1 and ME2 as coupled primers (Hales et al., 1996) (Table 1). Each amplification reaction was performed in 50 μl reaction mix containing: 10 μl of 5× GO Taq® DNA polymerase buffer (Promega), 5 μl of 25 mM MgCl_2_ solution (Promega), 1 μl of 10 mM dNTPs (Eurogentec), 0.2 μl of each primer at 100 μM and 0.24 μl of 5 U.μl^−1^ GO Taq® DNA polymerase (Promega). All amplifications were conducted in 30 cycles of denaturation at 94°C for 1 min, annealing for 1 min 30 s and extension at 72°C for 7min. The annealing temperature was set at 55 and 50°C for *dsrAB* gene and *mcr*A gene, respectively.

### Cloning, sequencing of 16S rRNA and functional genes, phylogenetic and statistical analysis

Prior to cloning, positively amplified PCR products were purified using NucleoSpin® Gel and PCR Clean-up kit (Macherey Nagel) according the manufacturer's instructions.

All of the 16S rRNA clone libraries were carried out with the TOPO XL cloning kit (Invitrogen) and functional gene clone libraries with the pGEM®-T cloning kit (Promega), both following the manufacturer's recommendations. Positive clones were processed for sequencing at GATC Biotech (Konstanz, Germany) using M13F primers. Sequences were imported into the BLAST nucleotide search program through the National Center for Biotechnology Information (NCBI website: http://www.ncbi.nlm.nih.gov/BLAST) to find closely related sequences within the GenBank database. The clone library 16S rRNA sequences were aligned, edited and analyzed using Bioedit version 7.1.3 software. Phylogenetic trees were constructed using the MEGA 5 program (Kumar et al., 2008). The robustness of the inferred topologies was tested using 1000 bootstrap resampling of the trees calculated on the basis of neighbor-joining algorithm (Saitou and Nei, 1987) using the Kimura two-parameter correction matrix (Kimura, 1980). All sequences more than 97% similar were considered to belong to the same phylotype (OTU) and were clustered together in the alignment (Schloss and Handelsman, 2004).

The sequence data reported in this study have been submitted to GenBank nucleotide sequence databases under accession numbers KC901750 to KC901834 and KC901560 to KC901725 for the *Archaea* and *Bacteria* gene sequences, respectively, and KC901726 to KC901749 for the *mcrA* gene sequences and KC901835 to KC901870 for the *dsrAB* gene sequences.

To examine the influence of the deployment time, depth or substrata type on both archaeal and bacterial diversity, we used the UniFrac computational tool (Lozupone et al., 2006). The habitats (defined by: the duration of incubation, the depth of incubation and the type of substrata) were clustered using the jackknife environment clusters analysis tool with 100 permutations.

### Geochemical analysis

Interstitial fluids from the colonization modules and deep seawater above the Mat Mound site (Dive 1770) were sub-sampled and stored as follows: 10 mL of fluid was used to measure pH at room temperature. For the analysis of dissolved major and trace elements, 30 mL of sample was filtered through 0.22 μm (Sterivex™, Millipore) membrane and stored at 4°C. For hydrogen sulfide analysis, 10 mL was filtered through a 0.45 μm (Sterivex™, Millipore) membrane and precipitated as ZnS in 25 mL evacuated septum vials containing 0.1g of Zinc Acetate (Sigma-Aldrich) and stored at 4°C. In the AISICS1 module pouch number 1, two immiscible fluids were recovered: a small amount of a buoyant liquid (about 5 mL) overlying a saline, seawater-like liquid (around 60 mL). Only the denser phase was treated as described above while the lighter phase, likely composed of hydrocarbons, was not processed further. Concentration of major elements was measured using Inductively Coupled Plasma-Atomic Emission Spectrophotometry (ICP-AES, Ultima 2, Horiba JobinYvon) while the concentration of trace elements was measured using High-Resolution ICP Mass Spectrometer (HR-ICP-MS, Element 2, ThermoFisher), both operated at the Pole Spectrometry Ocean Brest (PSO, Brest). Prior to elemental analysis, samples were acidified at least 1 month in advance to 0.28 mol.L^−1^ HNO_3_ prepared from ultra-pure reagent grades. Solutions for ICP-AES and ICP-MS analysis were diluted 100-fold with 0.28 mol.L^−1^ HNO_3_. Three water solution standards (Slew 3, Cass 4 and Nass 5 from the National Research Council of Canada) were also prepared along with the samples. For both ICP-AES and ICP-MS analysis, two sets of calibrating standards were used by adding multi-elemental standard solutions either with pure Milli-Q™ water or with 100-fold diluted Cass 4 in 0.28 mol.L^−1^ HNO_3_. Dissolved hydrogen sulfide was measured using spectrophotometric method using the protocol described by (Cline, 1969).

### Scanning electron microscopy: SEM

Scanning electron microscopy (SEM) was carried out at the “Service Commun de Microscopie Electronique à Balayage” (UPMC, Paris, France) using a Zeiss SUPRA® 55 VP Field Emission Scanning Electron Microscope (FE-SEM). The variable chamber pressure capability (2–133 Pa) permits the examination of both uncoated and Au- or C-coated samples. Three secondary electron detectors (Everhart-Thornley for high voltage mode, VPSE used for variable pressure mode and InLens for low voltage mode) and a backscattered electron detector enable the acquisition of high-spatial resolution images using analytical conditions that varied from 3–30 kV, 10 pA-1 nA, and 30–133 Pa with a 3.3–7.2 mm working distance. We also performed elemental microanalysis using an Energy Dispersive X-ray spectrometer (PGT Sahara).

### Confocal RAMAN spectroscopy

RAMAN spectra were obtained at IPGP (Paris, France) on resin free samples using a Renishaw InVia spectrometer. A 514 nm argon laser (20 mW) was focused through an Olympus BX61 microscope equipped with an x50 objective (numerical aperture 0.75). This configuration yields a planar resolution of about 1 μm, with a power delivered at the sample surface of 0.5 mW. An integration time of 100 s was used to ensure that the delivered radiation didn't damage the organic matter. The signal was dispersed using a holographic grating with 1800 grooves.mm^−1^ coupled for the detection with a RENCAM CCD (charge-coupled device) detector. The acquired RAMAN spectra were then processed using the WiRE 3.3 Renishaw software and compared to the RRUFF database (http://rruff.info/).

## Results

### Fluid geochemistry

About 60 mL of fluids were successfully recovered in each pouch of AISICS1, whereas very low quantities of fluid were pumped in AISICS2, probably due to clogging of the inlet. Hence, H_2_S and pH determinations were not performed for AISCIS2.

During the AISICS1 deployment, the average fluid temperature was 44.3°C with minimum and maximum values of 36 and 57.6°C, respectively. The fluid exhibited a near neutral pH (7.6) and low dissolved H_2_S concentrations (below 5 μM). For AISICS2, the average temperature was 42.9°C with a minimum at 36.9°C and a maximum at 46.3°C (Table 2). In general, the concentrations of major cations (Ca, K), trace metals (Mn, Fe), and Si were higher in AISICS1 compared to AISICS2 (Table 2), reflecting a higher contribution of hydrothermal fluids in the AISICS1 colonization module. This is consistent with the lower concentration of Mg in the AISICS1, which is typically depleted in hydrothermal vent fluids (Von Damm et al., 1985a,b). In general, fluids recovered from AISICS2 had chemical compositions quite similar to the overlying seawater (Table 2).

**Table 2:** **Geochemical composition and pH measured in the sampling pouches and bottom seawater (Dive 1770)**.

	**Mean T°C (max–min)**	**Pouch number**	**pH**	**H_**2**_S**	**Mg**	**Na**	**K**	**Ca**	**Sr**	**S**	**Si**	**Ba**	**Fe**	**Mn**	**Mo**
				**μM**	**mM**	**mM**	**mM**	**mM**	**mM**	**mM**	**mM**	**μM**	**μM**	**μM**	**μM**
AISICS 1	44.3 (57.6-36)	SX1-A	7.5	<5	42.0	434.0	13.8	13.8	0.13	29.66	1.88	0.96	0.88	17.83	<0.01
		SX1-B	7.6	<5	45.0	468.2	15.3	15.1	0.14	31.91	1.94	1.03	0.83	19.34	<0.01
		SX1-C	7.6	<5	44.5	466.2	15.1	15.0	0.14	31.21	1.82	1.02	1.06	18.46	<0.01
		SX1-D	7.6	<5	45.2	472.1	15.3	15.1	0.13	30.62	1.87	0.99	1.21	17.88	<0.01
AISICS 2	42.9 (46.3-36.9)	SX2-A	nd	nd	53.5	485.3	10.3	10.0	0.10	32.52	0.10	0.07	0.03	0.07	0.10
		SX2-B	nd	nd	53.6	482.7	10.5	9.8	0.10	33.16	0.11	0.07	<0.02	0.07	0.11
		SX2-C	nd	nd	55.1	492.9	10.9	10.2	0.10	32.58	0.18	0.11	<0.02	0.23	0.10
		SX2-D	nd	nd	53.2	481.1	10.4	9.8	0.10	32.75	0.16	0.10	<0.02	0.13	0.10
Bottom seawater (Dive 1770)			nd	nd	54.6	489.3	10.6	10.1	0.11	34.08	0.20	0.15	<0.02	0.17	0.12

nd for not determined.

Sulfate concentrations, determined as total dissolved sulfur on acidified and filtered sample (i.e., devoid of H_2_S) in AISICS1 and AISICS2 were close to seawater values, albeit slightly lower for AISICS1, consistent with the higher contribution of sulfate-depleted hydrothermal fluid. Additional evidence that the AISICS2 incubator was deployed under seawater dominated conditions comes from Mo concentrations (Table 2). Under anoxic conditions, where [H_2_S] ≥ 11 μ M and [O_2_] ≈ 0 μ M, seawater-derived molybdate ion will be reduced to the reactive tetrathiomolybdate species (Erickson and Helz, 2000) and readily precipitated. Hence, the complete removal of Mo observed in AISICS1 suggests predominantly anoxic, and probably sulfidic conditions while seawater-like Mo concentrations in AISICS2 provide evidence for rather oxic or micro-aerophilic conditions.

### Microbial diversity according to 16S rRNA genes sequences

**Figure 2:**
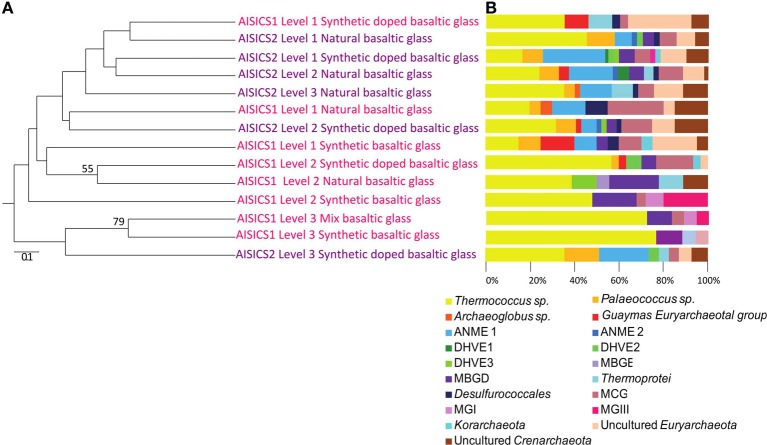
**Archaeal communities associated with the AISICS 1 and 2 mini-colonizers according the depth (i.e., position within the colonizer) and type of substratum for each colonization module. (A)** Jackknife environment cluster tree (made using the weighted UniFrac metric, based 16S rRNA gene sequences determined by neighbor-joining tree) showing the phylogenetic relationships among the archaeal lineages detected in each AISICS 1 and 2 mini-colonizers according the depth and substrata. The jackknife statistical analysis was done with one hundred replicates; the jackknife value was tagged near their corresponding nodes (values higher 50%). The scale bar corresponds, in the Unifrac unit, to the distance between the different habitats. **(B)** Proportions of archaeal groups within the clone libraries obtained from each AISICS 1 and 2 mini-colonizers.

**Figure 3:**
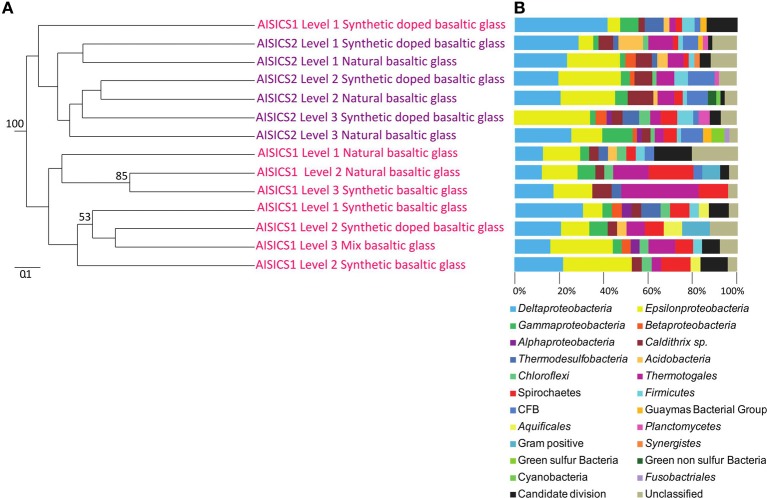
**Bacterial communities associated with the AISICS 1 and 2 mini-colonizers according the depth (i.e., position within the colonizer) and type of substratum for each colonization module. (A)** Jackknife environment cluster tree (made using the weighted UniFrac metric, based 16S rRNA gene sequences determined by neighbor-joining tree) showing the phylogenetic relationships among the bacterial lineages detected in each AISICS 1 and 2 mini-colonizers, according the depth and substrata. The jackknife statistical analysis was done with one hundred replicates; the jackknife value was tagged near their corresponding nodes (values higher 50%). The scale bar corresponds, in the Unifrac unit, to the distance between the different habitats. **(B)** Proportions of bacterial groups based on the frequency of 16S rRNA gene in clone libraries obtained from each AISICS 1 and 2 mini-colonizers.

The 16S rRNA gene was analyzed for 24–50 clones for each sample. High bacterial and archaeal diversity was generally observed in both colonizers with a slight difference in relation to the position of the mini-colonizers within the incubator (i.e., top or bottom). This translated into an increase in phylogenetic diversity with increasing depth in the sediment (Figures 2, 3) and Table 3).

**Table 3:**
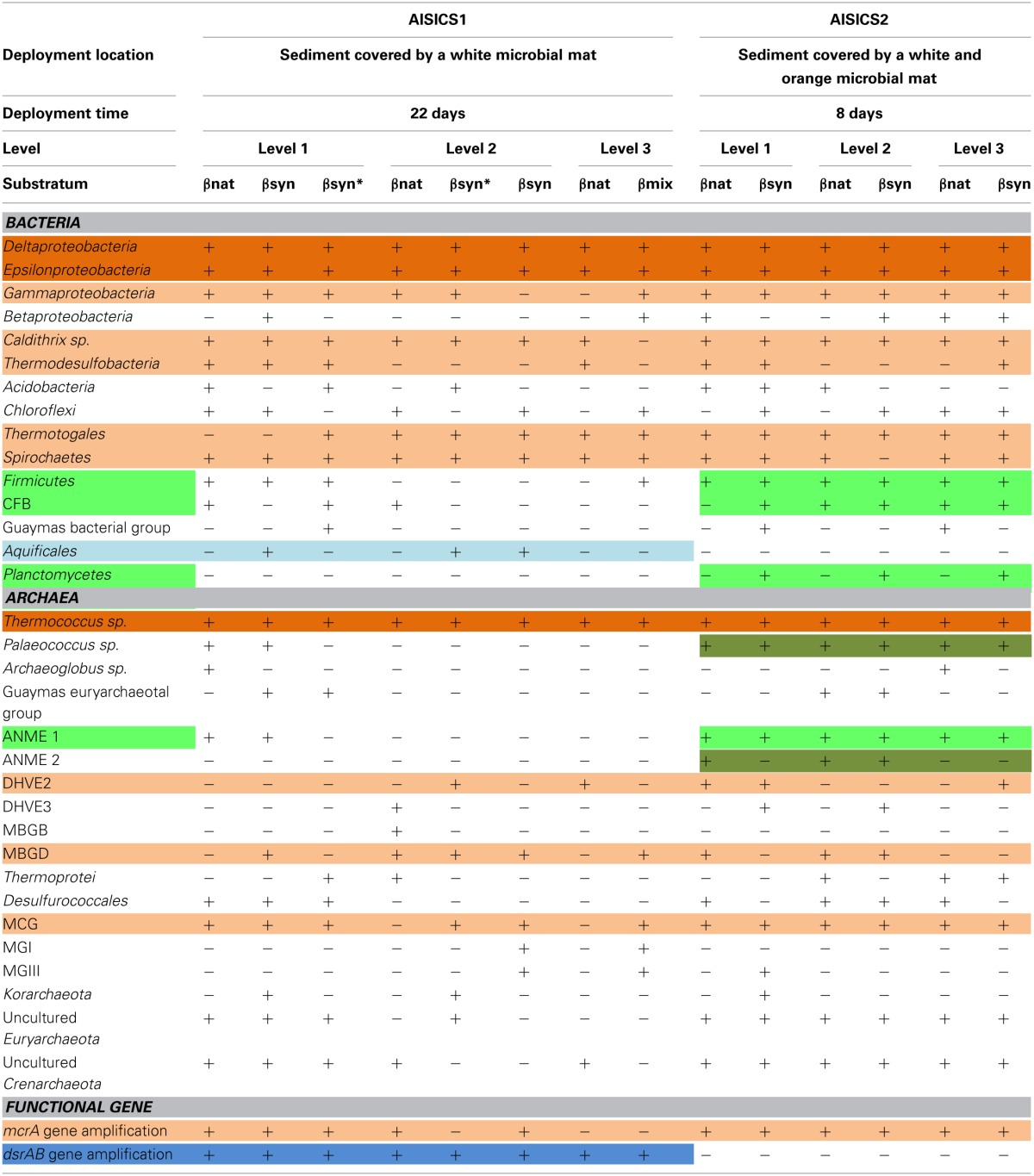
**Microbial composition determined per level, substratum and module**.

Only groups with more than 3 clones per samples are presented. + presence; − absence.

In dark orange, microbial group retrieved in all samples; in light orange microbial group retrieved in almost all sample.

In dark blue, microbial group retrieved only in all and/or mainly in AISICS1 samples; in light blue microbial group retrieved only in almost all and/or mainly in AISICS1 samples.

In dark green, microbial group retrieved only in all and/or mainly in AISICS2 samples; in light green microbial group retrieved only in almost all and/or mainly in AISICS2 samples.

**Figure 4:**
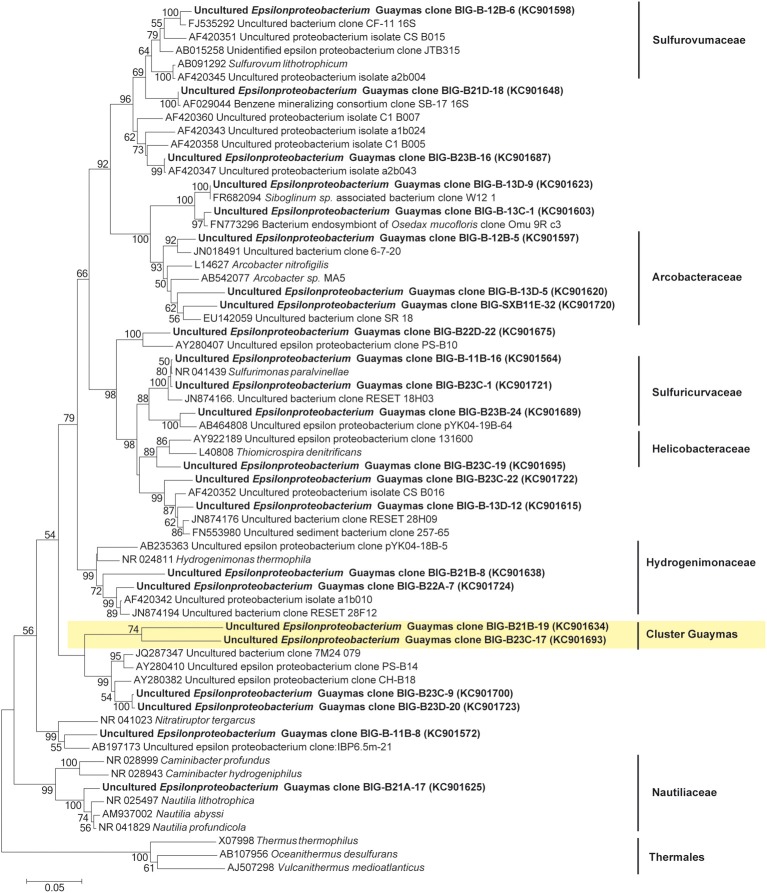
**Neighbor-joining phylogenetic tree of the *Epsilonproteobacteria*, based on the 16S rRNA gene sequences**. Bootstrap values above 50% (from 1000 bootstrap samples) are indicated near their corresponding nodes. In Yellow, the cluster of *Epsilonproteobacteria* cluster Guaymas; Thermales were used as outgroup.

In general, the main groups retrieved in all samples were the *Epsilonproteobacteria, Deltaproteobacteria*, and *Thermococcus* sp. In addition, *Gammaproteobacteria, Caldithrix sp*., *Thermotogales*, and *Spirochaetes* were observed in lesser proportions, and the DHVE2 (Deep-sea Hydrothermal Vent *Euryarchaeota* group 2) were also detected (Figures 2, 3 and Table 3). Sequences belonging to *Siboglinidae* as *Osedax sp*. or *Siboglinum sp*. endosymbiont and sequences close to the uncultured WS3 candidate division were retrieved in AISICS 1, the sampler that experienced a higher contribution of hydrothermal fluids and longer exposure time. In contrast, a new clade of *Epsilonproteobacteria*, named Guaymas *Epsilonproteobacteria* group (Figure 4), DHVE-1 (Deep-sea Hydrothermal Vent *Euryarchaeota* group 1) as well as ANME 2 sequences were found only in AISICS2 (Figure 2 and Table 3).

The cluster tree obtained with the Archaeal sequences (Figure 2) using the statistical jackknife environment clusters did not show any correlation between the archaeal diversity and deployment duration, the depth, or substrata composition. This contrasts with the cluster tree obtained for the Bacteria, where there was a correlation between bacterial diversity and deployment time and hydrothermal contribution (samples from AISICS1 and from AISICS2 were clustered together, respectively) and with depth in the sediment and the temperature for AISICS2 only (Figure 3).

### *mrcA* and *dsrAB* gene diversity

**Figure 5:**
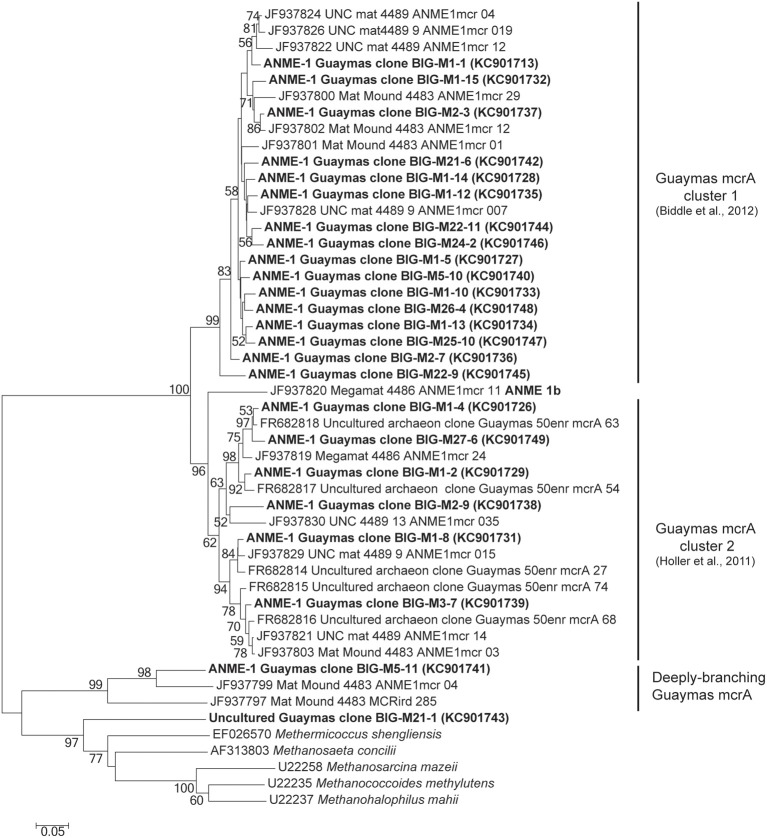
**Neighbor-joining phylogenetic tree of *mcrA* gene sequences**. Bootstrap values above 50% based on 1000 replicates are displayed.

The *mcrA* gene sequences were detected in AISICS1, in particular in the deepest mini-colonizers (Table 3). In AISICS2, the *mcrA* gene was amplified in almost all mini-colonizers irrespective of deployment depth. With the exception of one methanogen sequence detected in a mini-colonizer containing β nat substrate, all *mcrA* sequences were affiliated to ANME 1 related to the Guaymas *mcrA* cluster (Holler et al., 2011; Biddle et al., 2012) or to the deeply branching Guaymas mcrA cluster (Biddle et al., 2012) (Figure 5).

**Figure 6:**
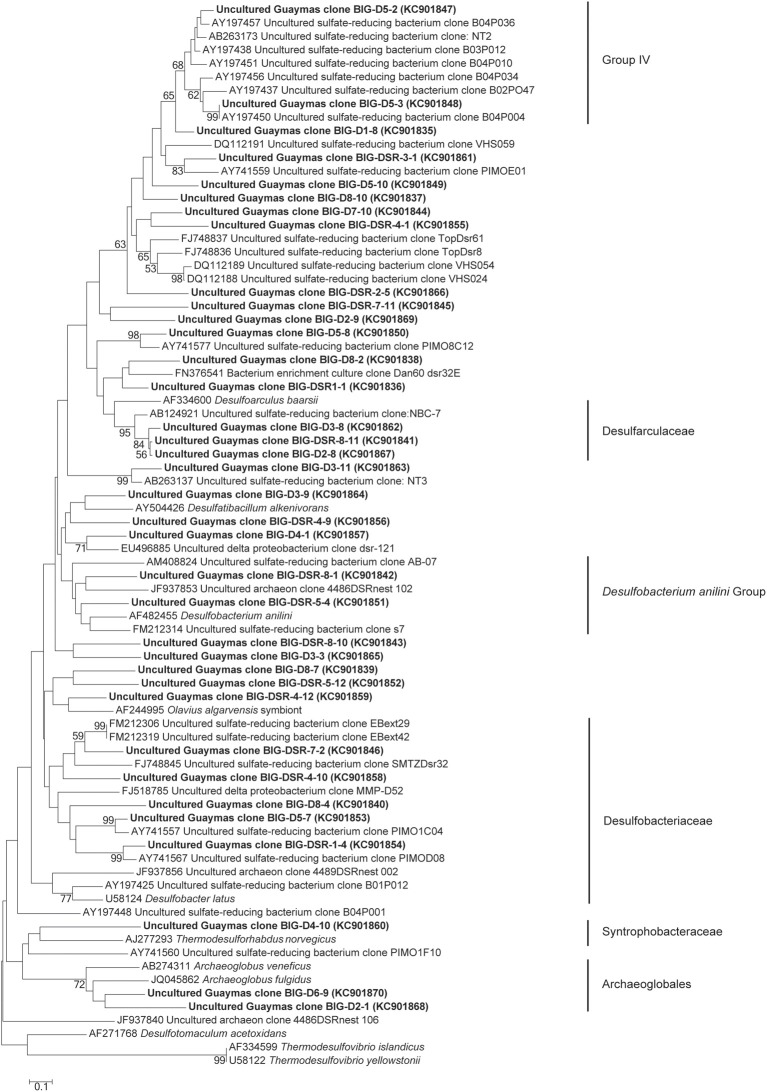
**Neighbor-joining phylogenetic tree of predicted amino acid translations of partial *dsrAB* gene**. Bootstrap values above 50% based on 1000 replicates are reported.

Using *dsrAB* gene sequencing, sulfate-reducers were detected in all mini-colonizers of AISICS1 (Table 3) but none in AISICS2. The majority of *dsrAB* (Figure 6) sequences were related to *Deltaproteobacteria*, especially the *Syntrophobacteraceae*, and some were close to *Desulfoarculaceae, Desulfohalobiaceae*, and *Desulfobacteriaceae, Desulfobacterium anilini* group and to group IV (Dhillon et al., 2003). In addition few *Archaeoglobus* sequences were found in most samples.

### Microscopy and raman spectroscopy analyses

**Figure 7:**
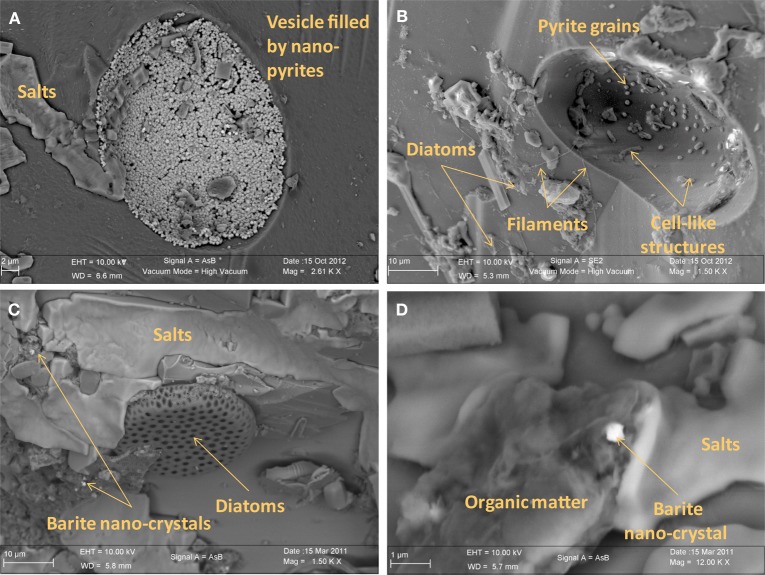
**Scanning electron microscopy photographs of basaltic glasses exposed to biotic conditions in AISICS1 module. (A)** vesicle filled with nano-pyrite on natural basaltic glass; **(B)** vesicle containing cell like structures and pyrite grains on natural basaltic glass; **(C)** heap of organic matter and diatoms with barite nano-crystals encrusted in organic matter; **(D)** magnified of organic matter heaps with barite nano-crystals surrounded by salts.

**Figure 8:**
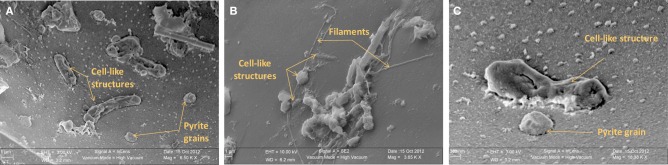
**Scanning electron microscopy photographs of natural basaltic glasses exposed to biotic condition in AISICS1 module showing in (A) cell-like structures and pyrite crystal inside a vesicle, in (B) cell-like structures, diatoms, and filaments at the glass surface and in (C) cell-like structure and pyrite crystal inside a vesicle**.

Irrespective of their composition (i.e., natural or synthetic) or exposure conditions (i.e., biotic or abiotic), microscopy analyses show that glass surfaces are covered by salt crystals (NaCl or MgCl_2_), and sulfate minerals (CaSO_4_· 2H_2_O gypsum or BaSO_4_ barite) due to direct precipitation from seawater after sample recovery. Glass surfaces from both AISICS modules did not present any clear alteration textures or replacement by secondary minerals. All natural glass fragments (β nat) and several artificial glass fragments (β syn or β syn^*^) have exhibited small rounded vesicles whose diameters vary between 10 and 100 μm (Figure 7). Those cavities were filled with sparse crystals of pyrite (Figure 7). In some cases, vesicles could be completely filled with nano-pyrite (Figure 7A). Since vesicles were present in β nat before deployment, they represent original features of submarine basalts that formed during magma degassing and were preserved during quenching. Interestingly, vesicles were not observed on the β syn and β syn^*^ before deployment. In addition to halite and pyrite crystals, vesicles of biotic samples also contain filaments and microbial cells-like structures. The biotic samples also exhibited an enrichment in organic matter forming small aggregates or film covering the glass surface (Figures 7, 8). In some cases, accumulations of organic matter with remnants of diatoms were observed together with framboidal pyrite or nano-crystals of barite (Figures 7, 8).

**Figure 9:**
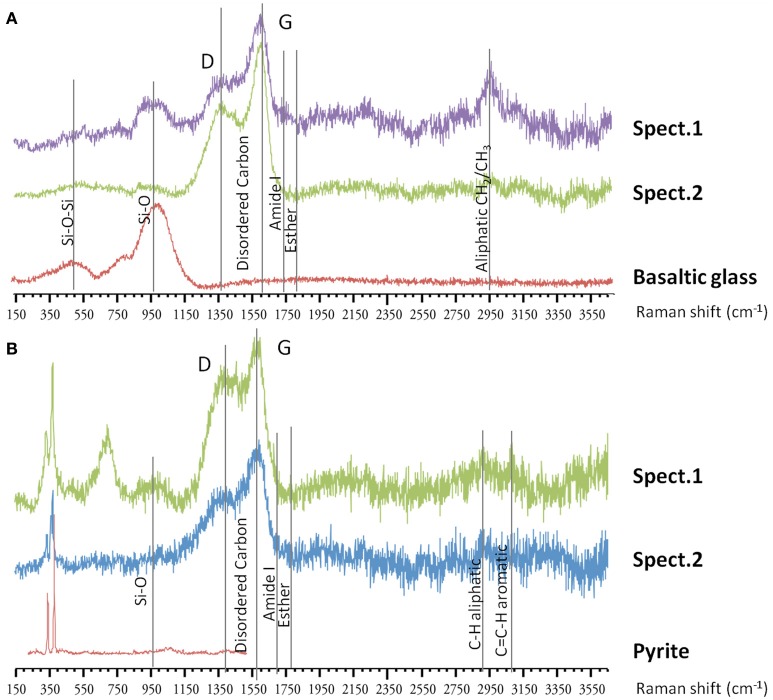
**Raman spectra on basaltic glasses exposed to biotic conditions. (A)** Raman spectra (spect.1 and spect.2) at the surface of ^57^Fe-doped synthetic basaltic glass showing the characteristic bands of disordered organic matter around 1360–1580 cm^−1^, along with aliphatic stretching between 2800–3000 cm^−1^, that could correspond to degraded microbial mat observed as aggregate at the surface. **(B)** Raman spectra (spect.1 and spect.2) inside a vesicle from natural basaltic glass showing similarly the presence of variably-degraded organic matter with typical bands around 1360–1580 cm^−1^, and between 2800–3000 cm^−1^ which could correspond to microbial mat, and two vibrational bands at 334 and 369 cm^−1^ assigned to pyrite.

In the associated Raman spectra, we observed broad and overlapping bands, designated as D and G bands (at 1360 and 1580 cm^−1^, respectively), along with the aliphatic and aromatic C-H vibrational bands between 2800–3000 cm^−1^, that are characteristic of disordered carbonaceous matter with a weak structural organization (Figure 9) (Spötl et al., 1998). This likely corresponds to degraded microbial mat as organic aggregates and microbial cells (mainly rods) were observed in both vesicles and on glass surfaces (Figures 7, 9) (Maquelin et al., 2002).

## Discussion

### Microbial colonization of basaltic glass

#### Microbial diversity and putative metabolisms

Miscellaneous groups of *Archaea* or *Bacteria* were detected in both short- and long-term deployments. According to the recorded temperature during incubation, all of the colonizing microbes should be mesophiles to thermophiles (Table 3), and exposed to mainly anaerobic conditions. In both colonization modules, archaeal and bacterial diversity generally increased with burial depth in the sediment (Figures 2, 3); this observation was more evident in the longer-term deployment module (AISICS1). The detected microorganisms could have several metabolisms including those involved in carbon, sulfur, iron, or nitrogen biogeochemical cycles. Although phylogenic affiliation may not be necessarily linked to specific metabolic or physiological properties, we cautiously inferred metabolic and physiological trends for clusters of microorganisms sharing similar properties. The implications of the observed microbial diversity for sulfur, iron, carbon, and nitrogen cycles are detailed below, with the aim to highlight potential biogeochemical reactions that may govern fluid-basalt interactions at high temperatures and in organic-rich environments:

***Carbon cycle.*** Due to the enrichment of organic matter at Guaymas basin, carbon cycling is likely a major metabolic driver in our colonizers. At Guaymas basin, the sediments accumulated a wide variety of organic compounds including light hydrocarbons, short-chain organic acids, particulate organic matter and ammonia (Welhan, 1988; Martens, 1990). These compounds were derived from diagenetic reactions between high temperature hydrothermal fluids and sediments, resulting in the pyrolysis of organic matter and precipitation of metal-sulfide in the subsurface. In biotic colonizers, organic compounds occur as small particle deposits or aggregates, droplets or mats, and result in characteristic RAMAN spectra (Figure 9). This organic matter, likely derived from the surrounding sediments, could directly support chemoorganotrophic microbial life associated with basalt substrata. We observed evidence for fermentative microorganisms (e.g., *Thermococcales*) that are likely involved in the degradation of complex organic substrates into smaller molecules such as short organic acids as acetate, amines, alcohol, H_2_, and CO_2_ (Orcutt et al., 2011b). Organic end products of fermentation, together with compounds resulting from pyrolysis processes, could be used by heterotrophic microorganisms detected in the AISICS1 and 2, such as those from CFB division, *Proteobacteria* or *Spirochaetes*. Organic acids could also be used as energy sources by a wide range of organotrophic microorganisms, including sulfate-reducing *Deltaproteobacteria*. In all cases, produced CO_2_ will be available for autotrophic microorganisms such as *Aquificales, Thermodesulfobacteria, Planctomycetes*, or some *Epsilonproteobacteria* that were detected in the modules. Methanogenesis may also occur, however, only one methanogen sequence was detected in the modules. In contrast, ANME phylotypes, which mediate anaerobic methane oxidation (AOM), were retrieved in almost all mini-colonizers from both AISICS modules. ANMEs involved in AOM in deep marine sediment are frequently associated with syntrophic sulfate-reducers, although nitrate, ferric iron, and manganese oxides may also serve as electron acceptors (Raghoebarsing et al., 2006; Beal et al., 2009). This issue is discussed in more detail in the following section.

In both colonizer modules, our microbial diversity surveys revealed the presence of both heterotrophic, autotrophic, and organotrophic microorganisms. These results suggest that anaerobic carbon cycling occurs in the colonizers in the same way as in the surrounding sediments. This finding is similar to studies of the microbial diversity of seafloor lava (Santelli et al., 2009) and Guaymas Basin sediments (Teske et al., 2009) but contrasts with ultramafic rock-hosted hydrothermal systems (Roussel et al., 2011) and pillow basalts (Mason et al., 2008; Santelli et al., 2008b), that are dominated by autotrophic organisms.

***Sulfur cycle.*** The data obtained from the 16S rRNA and dsrAB gene sequences both suggest that sulfate-reduction occurs, particularly due to the presence of members of the *Deltaproteobacteria, Firmicutes, Thermodesulfobacteria*, and *Archaeoglobales* (Figure 2; Table 3) (Widdel et al., 1992). Sequences of *Deltaproteobacteria* are found in all mini-colonizers, while dsrAB gene amplification was successful only in the long-term deployment (AISICS1), suggesting that in AISICS2 *Deltaproteobacteria* were not all sulfate-reducing bacteria. Indeed, strains belonging to the *Deltaproteobacteria* and the *Firmicutes* phyla are associated with numerous metabolisms in addition to sulfur metabolisms (Orcutt et al., 2011a,b). Microbial sulfate reduction has also been previously reported in Guaymas sediments (Dhillon et al., 2003; Teske et al., 2003; Biddle et al., 2012) and may occur in the colonizers using dissolved organic substrates and seawater sulfate. As discussed below, *in situ* sulfate reduction may also explain the occurrence of pyrite observed in basalt vesicles.

Sulfur-reduction is also inferred from the occurrence of *Epsilonproteobacteria, Desulfurococcales, Thermotogales, Thermococcales* as well as *Deltaproteobacteria* and *Planctomycetes* that were retrieved in all samples. Indeed, some isolated strains of these groups are able to reduce diverse sulfur compounds (Bertoldo and Antranikian, 2006; Campbell et al., 2006; Elshahed et al., 2007).

Based on the physiology of the isolate *Caldisericum exile*, which is a thermophilic, anaerobic, thiosulfate-reducing bacterium and affiliated with OP5 clones (Mori et al., 2008, 2009), and based upon the OP5 occurrence in sediments and sulfur-rich environments (Hugenholtz et al., 1998; Teske et al., 2002), it can be assumed that OP5 members could be also involved in sulfur cycle. A metagenomic study of OP3 division members suggested that they share similar metabolic properties with *Deltaproteobacteria* (Glöckner et al., 2010) and single-cell analyses revealed that SKK-01 strain harbors sulfur-containing intracellular inclusions (Kolinko et al., 2012). The physiological properties of *Aciduliprofundum boonei*, and the environmental niches of other DHVE2 members, demonstrate that this clade is highly involved in the sulfur cycle (Nercessian et al., 2003; Reysenbach et al., 2006; Flores et al., 2012). Thus, even if the physiological properties of these microorganisms still remain unclear, OP5, OP3, and DHVE-2 members could have played a role in sulfur cycle. Therefore, we suggest that an active anaerobic sulfur cycle took place within the mini-colonizers where both sulfate and sulfide coexist.

***Iron cycle.*** Considering the abundance of iron in volcanic glass and its potential importance for supporting endolithic microbial growth [e.g., (Bach and Edwards, 2003)], it is crucial to evaluate the role of microorganisms in iron biogeochemical cycling. Among the groups identified in our experiments, *Beta-* and *Alpha-proteobacteria, Thermotogales*, DHVE2, and OP3 members could all be involved in iron cycling. For example, within the *Thermotogales* (Vargas et al., 1998), and within the DHVE2 [*Aciduliprofundum boonei* (Reysenbach et al., 2006)], some species are able to grow as dissimilatory iron reducers using poorly crystalline ferric iron [Fe(III)] as an electron acceptor. In addition, *Betaproteobacteria* and some *Alphaproteobacteria* are able to oxidize Fe(II) (Edwards et al., 2003b; Nakagawa and Takai, 2008). Moreover, despite the lack of any cultivated OP3 members, the SKK-01 strain is a magnetotactic bacteria harboring Fe-containing magnetosomes (Kolinko et al., 2012). In addition, the OP3 group frequently occurs in anoxic deep-sea hydrothermal system and in heavy metal contaminated sediments (Teske et al., 2002; Rastogi et al., 2011), which may implicate OP3 in iron cycling.

The high concentration of dissolved Fe in AISICS1 may have multiple sources, including a direct contribution from hydrothermal fluids and dissimilatory iron reduction (DIR). High concentrations of other elements typically enriched in hydrothermal fluids (e.g., Si and Mn) argue for the former hypothesis and preclude identifying geochemical evidence for active DIR in the colonizers. In turn, both the prevailing anoxic conditions and our diversity surveys suggest the predominance of iron-reduction over Fe-oxidation pathways.

***Nitrogen cycle.*** The chemical analysis of the ambient fluid sampled through the colonizers (Table 2) showed an important seawater contribution of nitrate and nitrogen compounds which could have supported the growth of microorganisms in the colonizers. Our diversity survey corroborates previous studies demonstrating that denitrification took place in deep-sea sediments affected by hydrothermal circulation in the Guaymas Basin (Bowles et al., 2012). Nitrate is a common electron acceptor used by a number of microorganisms under anaerobic conditions (Brandes et al., 2007; Jetten, 2008). Among all the microorganisms known to be able to use nitrates as final electron acceptor, *Aquificales* (Gotz et al., 2002; Huber et al., 2002), *Firmicutes* (L'Haridon et al., 2006), *Caldithrix* (Miroshnichenko et al., 2003), and *Epsilonproteobacteria* (Bowles et al., 2012) were detected in both AISICS modules.

In addition, it appears that ANAMMOX bacteria may also be active in our colonizers. Sequences closely related to *Planctomycetes* were found in AISICS2 colonizers (short exposure time). Within the *Planctomycetes*, the ANAMMOX bacteria are the sole group known to be able to perform anaerobic oxidation of ammonium, (Jetten et al., 2005; Francis et al., 2007) where nitrite, one of the product of denitrification, serves as electron acceptor to form dinitrogen (gas) (Strous et al., 1999; Francis et al., 2007). Although the presence of sequences affiliated to *Planctomycetes* does not allow us to infer their function, ANAMMOX bacteria were known to be active in hydrothermal systems (Byrne et al., 2009) and were already detected in Guaymas basin sediment samples (Russ et al., 2013). Hence, all together, these results suggest that the anaerobic nitrogen cycle, denitrification, and ANAMMOX processes might all occur in our colonizer modules, and by extension, in the surrounding sediments. This finding suggests that the anaerobic part of the nitrogen cycle is one of major processes in hydrothermal sediments, as well as previously noted for basaltic substrates (Mason et al., 2008; Santelli et al., 2009).

***Uncultivated lineage and under-represented groups.*** Many sequences belonging to uncultivated lineages were detected. The lack of information about their putative physiology did not allow us to infer their role in the colonization process or their ecological importance. Members of the Guaymas Bacterial Group (Teske et al., 2002) and Guaymas *Euryarchaeotal* Group (Teske et al., 2002; Dhillon et al., 2005) were found in the two modules. These groups were previously detected in hydrothermally-affected deep-sea sediments and active chimneys of the Guaymas Basin (Teske et al., 2002; Callac et al., submitted). Since their distribution is restricted to hydrothermal environments, it can be assumed that these microorganisms are anaerobes and probably involved in organic matter and hydrocarbon compound degradation.

A new cluster of *Epsilonproteobacteria*, named Guaymas *Epsilonproteobacteria* group, was identified in AISCIS 2; this group is only 91% similar to any known environmental clones or cultivated representatives (Figure 4). Like other members of the *Epsilonproteobacteria* from hydrothermal ecosystems, these microorganisms could be mesophilic or moderately thermophilic and involved in organic matter degradation and sulfur cycling in organic matter-rich hydrothermally affected sediments.

#### AOM: ANMEs, potential syntrophs, and other members

ANME-1 and more specifically “ANME-1 Guaymas *mcrA* cluster” sequences (Holler et al., 2011; Biddle et al., 2012), as well as “deeply-branching Guaymas *mcrA”* sequences, were retrieved in both modules (Figure 5; Table 3). Most of them are affiliated to sequences previously found in Guaymas hydrothermal sediments with a range of temperature regime (Biddle et al., 2012; Merkel et al., 2013).

Interestingly, no *dsrAB* genes could be amplified from the AISICS2 module (short-term deployment) where ANME sequences were retrieved (Table 3). In contrast, both ANME and *dsrAB* sequences were detected in the long-term AISICS1 deployment that experienced a greater hydrothermal fluid contribution (Table 2). In addition to *Desulfobacteriaceae*, sulfate-reducers such as *Deltaproteobacteria* are known to be ANME syntrophs. However, none of those groups could be detected using either *dsrAB* (Figure 6), or 16S rRNA sequencing. This suggests that detected ANME might have other syntrophs. For example, sulfate-reducers identified in AISICS 1 such as *Syntrophobacterales, Desulfobacterium anilini* group, group IV or archaea *Archaeoglobus* could play this role. Another hypothesis is that the syntrophs are not sulfate-reducers but rather are denitrifiers or iron-reducers (Raghoebarsing et al., 2006; Beal et al., 2009). Potential syntrophs identified in most mini-colonizers could be *Thermotogales* involved in iron-reduction, or *Epsilonproteobacteria* and/or *Caldithrix* involved in nitrate-reduction. It is also possible that sulfate-reducers involved in AOM colonize AISICS modules after ANME, or that sulfate-reducers progressively replace other syntrophs (e.g., nitrates and/or iron-reducers) to create new consortia with ANME. Alternatively, we cannot exclude that the ANME, especially the AISICS2 ANME-1, are able utilize carbon, energy sources, and electron acceptor needed for their growth without syntrophs, as previously shown (Knittel et al., 2005), or by doing AOM alone (Milucka et al., 2012). In addition, within the *Archaea*, MCG sequences were detected. The MCG are well-represented in the deep subsurface biosphere (Sorensen and Teske, 2006; Teske and Sorensen, 2007; Kubo et al., 2012). In previous works, it was largely hypothesized that MCG are anaerobes and heterotrophs able to use organic substrates (Biddle et al., 2006). It has also been suggested that they are able to oxidize methane without the assimilation of methane-derived carbon, using dissimilatory methane metabolism (Biddle et al., 2006). They could also benefit from AOM, directly or not (Sorensen and Teske, 2006). In our colonization modules, MCG could play a direct or indirect role in the methane cycle in association with methanogens and ANMEs. These data support the idea that anaerobic methane cycling is common in hydrothermal systems (Teske et al., 2002).

#### Sediments: a nest for free-living symbionts?

Sequences of endosymbionts *of Siboglinidae* (*Osedax sp*. and *Siboglinum sp*., Figure 3) were retrieved in the AISCIS1 module. Previous studies have reported free-living symbionts in bottom seawater overlying seafloor hydrothermal fields (Harmer et al., 2008), or in microbial mats (Crépeau et al., 2011). At Mat Mound site, vent fauna include *Riftia* worms, an unidentified *Siboglinidae*, polychaetes *Paralvinella* sp. and *Ampharetidae* in association with microbial mat (Figure 1; Decker et al., pers. commun.). To date, symbionts of *Riftia* sp. and *Paralvinella* sp epibionts were never reported in their free-living form. However, it has been suggested that vent fauna may gain their endosymbionts locally, leading to an opportunistic environmental acquisition of the best adapted microorganisms (Rodrigues et al., 2011). The presence of free-living symbionts in hydrothermally-affected sediment (e.g., average temperature around 44.3°C) suggest they are able to live in such conditions, which highlights the role of sediment substrate for the dispersion and horizontal transmission of vent fauna symbionts.

### Microbial diversity and potential control of geochemistry, substrata type, temperature, and/or deployment time

A large microbial diversity was evident in the colonization modules, and some phylotypes were common among both modules. Archaeal diversity was not correlated with deployment duration, fluid chemistry, sediment depth, or substrata (Figure 2). In contrast, bacterial colonization patterns are driven by a number of factors, such as the duration of deployment and fluid chemistry (Figure 3). In AISICS 2, the bacterial diversity is also influenced by the burial depth in the sediments (Figure 3). This is supported by the statistical analyses of the 16S rRNA sequences for AISICS2, where the diversity clusters according to burial depth (Figure 3). In addition, the bacterial diversity tends to increase with depth (Figure 3), suggesting that the bacterial distribution could be linked to the thermal gradient and the availability of hydrothermally-derived compounds. A recent study at Guaymas Basin has shown that the thermal regime and geochemistry of hydrothermally-affected sediments are highly heterogeneous (Mckay et al., 2012). AISICS1 was deployed in a white mat and AISICS2 at the junction between white and orange mats, and while the *in situ* temperature at 20 cm depth was almost the same for both modules (average of 44.3 and 42.9°C, respectively), the geochemistry of recovered pore water was drastically different. Indeed, the long-term deployment module (AISICS1) experienced a much higher hydrothermal contribution than AISICS2 (Table 2).

This suggests that AISICS1 micro-colonizers encountered significant concentration of H_2_S (although <5 μ M) while fluids sampled in AISICS2 correspond mainly to heated seawater (Table 2). Hence, the geochemical differences between AISICS1 and 2 could explain the differences in bacterial colonization patterns. From the statistical jackknife environment clusters trees (Figures 2, 3), it is clear that microbial colonization is not related to other parameters such as substrata composition.

Given the high concentration of organic matter in the sediment (between 2 and 4% of organic carbon (Kastner, 1982), and the ubiquitous deposition of organic matter on basaltic glass surfaces, as observed by RAMAN spectroscopy and SEM, it is likely that heterotrophic strains could have been pioneers. Fermentative strains can hydrolyze organic matter into small compounds, e.g., small organic acids, amines, or alcohol. These metabolic products could have fueled other heterotrophs and organotrophs, as well as lithoautotrophs.

### Geomicrobiological interactions

**Figure 10:**
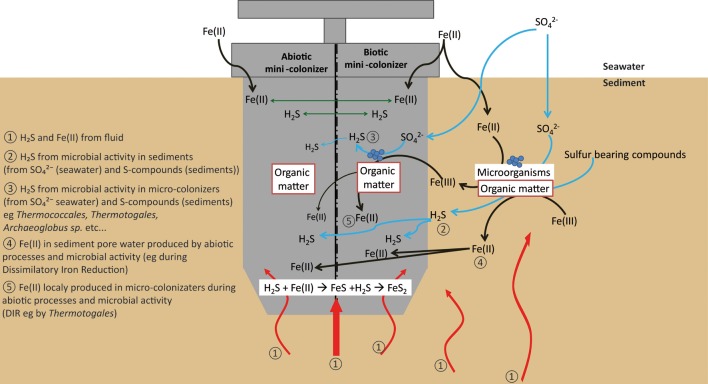
**Schematic diagram showing the different pathways for pyrite formation in both biotic and abiotic mini-colonizers**.

Both biotic and abiotic mini-colonizers were filled with identical substratum and exposed to the same environmental conditions allowing a direct comparison between chemical (abiotic) and biological processes taking place on the surface of basaltic glasses. To our knowledge, the systematic use of a sterile control for *in situ* basalt and/or mineral alteration experiments has never been attempted. Micro- and nano-crystals were observed, thus, it is important to note the difference between the micro- and the nano-crystal formation (both pyrite and barite). Micro-crystals of pyrite and barite were observed on basaltic glass surfaces incubated under both biotic and abiotic conditions (Figures 7, 8) suggesting that they result solely from inorganic processes. Similar to our results, laboratory microcosm experiments of basalt alteration have failed to reveal differences of alteration textures and secondary mineral precipitation between biotic and abiotic conditions (Einen et al., 2006). Nano-crystals of pyrite were only observed in basalts exposed to biotic conditions, which suggests a role of biological process in pyrite formation (Figures 7, 8). Although pyrite can precipitate abiotically from H_2_S and Fe^2+^ enriched in the hydrothermal fluid, microbial sulfate, and sulfur reduction could have promoted nano-crystalline pyrite precipitation instead of micro-crystalline pyrite (Figure 10). In addition, SEM observations of glass vesicles on biotic samples show a dense mineralization of nano-crystals of pyrite lining the cavity, and wrapped in a film of organic material. The vesicles likely provide a favorable microenvironment for pyrite precipitation, for example through local build up of microbially produced hydrogen sulfide, leading to supersaturation with regard to pyrite (or its FeS mono-sulfide precursors, Berner, 1984). Framboidal pyrite mineralization was also observed on biotic glass surfaces. Although initially attributed to microbial process, this type of pyrite may form without microbial activity (Butler and Rickard, 2000). Ongoing study of sulfur isotopes of pyrite should help in distinguishing between those two models [e.g., Canfield, 2001; Rouxel et al., 2008a,b].

Nano-crystals of barite were also observed in close association with organic matter, suggesting similarly a possible biological mediation for nano-barite crystallization. Barite is known to form bio-aggregates in association with decaying organic matter (Bishop, 1988). However, a direct precipitation of barite from hydrothermal fluid is also possible due to the enrichment of Ba in hydrothermal fluids (Von Damm et al., 1985b).

Small rounded to slightly elongated vesicles (10–100 μm diameter; Figure 7) were observed in all glass samples. The occurrence of vesicles in synthetic glass implies that they existed before incubation, although they were not detected macroscopically due to their small size of less than 50 μm. As for natural basaltic glass, vesicles formation is likely due to gas micro-bubbles in silicate melt, trapped, and preserved as vesicles during quenching.

Accumulations of diatom debris and carbon-rich aggregates were frequently observed on biotic samples (Figures 7, 8) but carbon-rich aggregates were also identified on abiotic samples due to the quantity of organic matter present in the neighboring sediments. The presence of microbial cells has been nonetheless observed using SEM imaging only on biotic samples. Finally, in comparison with studies done on long time (1 year; Einen et al., 2006) or on natural samples (Thorseth et al., 1992; Furnes et al., 2001) the lack of glass alteration evidence is certainly due to the relatively short deployment time (less than 22 days), precluding formation of even incipient alteration rims.

## Conclusion

By deploying *in-situ* colonization modules this study showed that diverse microbial communities involved in carbon, nitrogen, sulfur, and iron cycles are able to colonize the surface of basaltic glasses in hydrothermal and organic matter-rich conditions.

While the archaeal colonization pattern is not dependent upon deployment duration, fluid chemistry, sediment depth, or substrata composition, the diverse bacterial colonization patterns are driven by deployment time and fluid chemistry. In all cases, the nature of basalt does not seem to influence microbial colonization.

The presence of a new cluster of *Epsilonproteobacteria*, the Guaymas *Epsilonproteobacteria* group, which is distantly related to any known environmental clones and cultivated representatives expand our current view of microbial diversity in hydrothermal systems.

In some cases, we detected anaerobic methane oxidizers related to ANME 1 and 2, which were not associated with their usual sulfate-reducer syntrophs. This suggests that the ANME groups detected in this study are able to live without syntrophs or may have other sulfate-reducer, denitrifier (some *Epsilonproteobacteria* and/or *Caldithrix*), or iron-reducer (*Thermotogales*) syntrophs.

Despite the lack of specific glass alteration textures, the formation of secondary minerals was observed on glass surface for both biotic and abiotic experiments. Micro- and nano-crystalline pyrite was generally detected within basalt vesicles associated with organic matter aggregates. Further work, for example applying sulfur isotope systematic, is required to discriminate between biotic and abiotic processes involved in pyrite formation. Applying a similar experimental approach in future studies, providing that deployment duration is sufficient, should provide new insights into the capability of microbial communities to exploit new environmental conditions, colonize new niches, and promote mineral and rock substrate alteration.

### Conflict of interest statement

The authors declare that the research was conducted in the absence of any commercial or financial relationships that could be construed as a potential conflict of interest.
